# Population Ecology of Free-Roaming Cats and Interference Competition by Coyotes in Urban Parks

**DOI:** 10.1371/journal.pone.0075718

**Published:** 2013-09-13

**Authors:** Stanley D. Gehrt, Evan C. Wilson, Justin L. Brown, Chris Anchor

**Affiliations:** 1 School of Environment and Natural Resources, Ohio State University, Columbus, Ohio, United States of America; 2 Max McGraw Wildlife Foundation, Dundee, Illinois, United States of America; 3 Forest Preserve District of Cook, County, Elgin, Illinois, United States of America; University of Sydney, Australia

## Abstract

Free-roaming cats are a common element of urban landscapes worldwide, often causing controversy regarding their impacts on ecological systems and public health. We monitored cats within natural habitat fragments in the Chicago metropolitan area to characterize population demographics, disease prevalence, movement patterns and habitat selection, in addition to assessing the possible influence of coyotes on cats. The population was dominated by adults of both sexes, and 24% of adults were in reproductive condition. Annual survival rate was relatively high (S=0.70, SE=0.10), with vehicles and predation the primary causes of death. Size of annual home range varied by sex, but not reproductive status or body weight. We observed partitioning of the landscape by cats and coyotes, with little interspecific overlap between core areas of activity. Coyotes selected for natural habitats whereas cats selected for developed areas such as residences. Free-roaming cats were in better condition than we predicted, but their use of natural habitat fragments, and presumably their ecological impact, appeared to be limited by coyotes through intraguild competition.

## Introduction

Because of their association with humans, domestic cats (*Felis catus*) are one of the most widely-distributed terrestrial mammals. Within cities, domestic cats may be the most abundant mesocarnivore in some parts of the urban landscape [1,2]. This seeming omnipresence of free-ranging cats that either may be owned or semi-feral has led to controversies [3,4]. These issues are well documented, but briefly free-roaming cats have been reported to depredate native wildlife and, in some instances, appear to have reduced or even extirpated some populations [5,6]. Also, there is a perception that free-ranging cats are often in poor health [7], and cats may spread pathogens for which they are host, or in other cases may facilitate the transmission of other pathogens to sympatric wildlife or people [3]. However, data on the population ecology of free-ranging cats, and especially aspects that relate to potential predation or disease risk, are needed. This information gap is especially true for cats inhabiting urban landscapes, where their numbers can reach inordinately high levels and the systems are already stressed from other anthropogenic effects [1].

Individual cats vary in their reliance on people for resources; likewise, humans vary in the level of support provided to cats [1]. Some cats are sterilized, vaccinated, and provided for by cat advocates as part of feral cat management programs, and others may be treated or not by owners that provide varying amounts of attention or care. However, demographic and disease profiles of the cat population are needed to assess the direction and scope of management programs, whether they are removal programs or Trap-Neuter-Release (TNR) programs.

Despite their seeming ubiquity across urban landscapes, ecological impacts presumed to occur from free-ranging cats may be mitigated by larger mammalian carnivores [5]. In North America, this largely involves the coyote ( *Canis* *latrans* ), as this species has expanded into most metropolitan areas [8]. The mesopredator release reported by Crooks and Soule [5] was largely driven by the coyote-cat relationship, in which they provided correlative evidence of intraguild competition between the species. However, the extent to which coyotes influence cat numbers or movements, and the mechanism(s) responsible, have not been rigorously assessed for any system.

We focused our research on free-roaming cats inhabiting patches of natural habitat within the larger Chicago metropolitan area. Our objectives were to: 1) report the demographic characteristics of the free-roaming cat population, especially regarding a possible linkage between physical condition and sterilization, 2) to identify the prevalence of diseases associated with cats, 3) estimate survival and identify causes of mortality, 4) to determine movement patterns of cats and habitat selection, and 5) compare the space use of cats to that of coyotes in those areas of sympatry and where we monitored both species concurrently. We were specifically interested in whether cats avoided areas or habitats used heavily by coyotes.

## Methods

### Study Area

**Figure 1:**
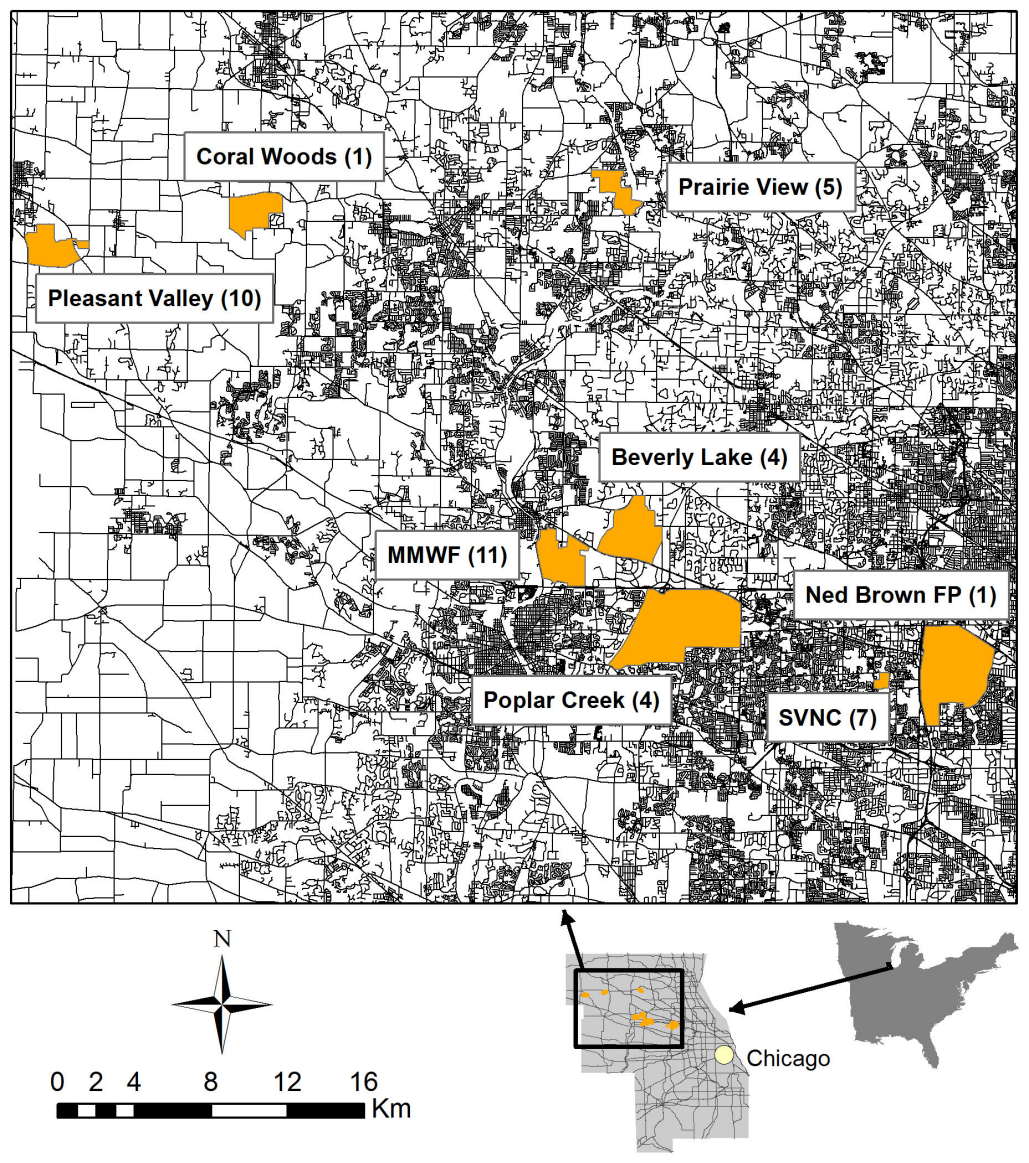
Capture locations of free-roaming cats in the Chicago metropolitan area. Study sites are referenced in orange with the samples sizes indicated at each site (n). All cats were sampled during 2008-2009. Black lines indicate primary, secondary and residential roads as an indicator of urbanization.

The Chicago metropolitan area includes >260 municipalities and a cumulative human population exceeding 8 million, making it one of the largest urban centers in the United States [9]. Our work took place at multiple sites within the northwestern suburbs of the Chicago metropolitan area (Figure 1). The sites were natural areas and were selected based partially on our concurrent work on urban coyotes [8,10] and where there were reports of unowned free-ranging cats on site. Our trapping sites were the Max McGraw Wildlife Foundation in Kane County, Illinois, the Poplar Creek Forest Preserve, Beverly Lake Forest Preserve, Ned Brown Forest Preserve, and the Spring Valley Nature Preserve all in Cook County, Illinois, and Prairie View Conservation Area and Pleasant Valley Conservation Area, located in McHenry County, Illinois. The Max McGraw Wildlife Foundation is a private property, whereas the other sites are public parks or conservation areas.

### Livetrapping

We conducted livetrapping opportunistically at these sites during March 2008 through November 2009. When putative free-ranging cats were reported at these sites, we placed box livetraps (81 X 25 X 30 cm, model 108, Tomahawk Live Trap Co., Tomahawk, Wisconsin) at sites deemed to maximize trapping success, and were maintained until cats were captured. Traps were baited with commercial canned cat food and checked each morning and, at some sites, also in the evening.

Upon capture, cats were inspected to determine the likelihood they were owned. In reality, the degree to which a cat is ‘owned’ is not necessarily a discreet characteristic, but we attempted to focus only on cats that were not clearly owned. We determined ownership status based on several characteristics including, an individual’s physical state (i.e. fitness, cleanliness), presence of a PIT (Passive Integrated Transponder) tag or collar, presence of an ear notch or clip (which indicates sterilization) and the behavioral response of an individual to people (i.e. tameness). The Spring Valley Nature Center differed from the others in that there was a managed cat colony present, and we captured cats associated with that colony. However, other sites may have had TNR colonies nearby that we were unaware of, and some of the cats we captured may have had loose associations with colonies. We did not sterilize cats as part of the project due to concerns over capturing an owned cat, and due to the fact that observing differences between sterilized and unsterilized cats was an important objective.

Cats that we deemed to be ‘feral’ were chemically immobilized with a 10 mg intramuscular injection of Telazol® (Fort Dodge Animal Health, Fort Dodge, Iowa). For each cat, we recorded body weight, linear body measurements (right ear length, right foot length, tail length, and total length [body + tail]), reproductive condition (presence of testes or neuter scar), age (juvenile or adult). Pelage coloration and other physical characteristics were recorded. We also attempted to collect blood samples for serology tests. We drew approximately 2-3 ml of blood from the jugular or lateral saphenous veins. Adult cats and juvenile cats > 2.5 kg were fitted with VHF radiocollars (Advanced Telemetry Systems, Isanti, Minnesota, USA). Collars weighed approximately 33 g and were fitted with trailing antennae. Cats were allowed to recover from immobilization and released at, or near, the capture site in the evening.

### Serology

We placed blood samples into separator tubes, allowed them to clot, and then centrifuged and separated the samples. The serum was then removed from each sample and transferred to microcentrifuge tubes and frozen at -70°C. Serologic testing was performed at the University of Illinois Veterinary Diagnostic Laboratory (Champaign, Illinois, USA). We collected subsequent samples from individual cats only if they were recaptured in a different season or year. We used serology to determine the prevalence of feline immunodeficiency virus (FIV), feline leukemia virus (FeLV), *Toxoplasma gondii*, and feline heartworm among free-ranging cats. These consisted of ELISA tests for FIV, FeLV, and heartworm. The ELISA tests for FIV and heartworm detect the presence of antibodies, whereas the test for FeLV detects the presence of proteins associated with the virus. Tests for the presence of antibodies for *T. gondii* were performed using the indirect immuno-fluorescence (IFA) slide test, using ImmunoFA product No. 1207 toxoplasma slides (GenBio, San Diego, California, USA). A titer of ≥ 1:40 was considered positive for exposure to *T. gondii* for IgG and IgM immunoglobulins. The presence of IgM antibodies typically indicate a recent or active infection, while IgG antibodies reflect an infection in an individual’s past [11]. FIV and FeLV are typically directly transmitted between individuals, whereas individuals are typically infected with *T. gondii* and feline heartworm through environmental pathways.

### Radiotracking

Radio-collared cats were located via trangulation from truck-mounted antenna arrays. Free-ranging cats are typically more active at night [12] therefore we only recorded one location during daytime hours and conducted sequential monitoring, with a minimum of one hour between relocations, of cats at night. The number of nights cats were tracked varied depending on time of year, but we maintained similar numbers of nights among seasons. We obtained radiolocations for cats by visual observations, triangulation with program LOCATE III (Pacer, Truro, Nova Scotia, Canada), or by circling the animal’s location with a truck-mounted antenna and record their location directly with a Global Positioning System (GPS) unit. Triangulations were recorded using a minimum of three bearings with a maximum of twenty minutes between first and final bearings. Location error was estimated to be 141.2 (41.9) m using test collars. The latter was possible when cats moved into the urban matrix and the road system allowed us to closely follow animals. Cat locations were recorded to the nearest meter using the Universal Transverse Mercator (UTM) grid system.

Radiotransmitters also included a mortality switch and we attempted to collect carcasses of cats as quickly as possible, usually within 24-48 hours, following indications of mortality. We submitted cat carcasses to the University of Illinois Zoological Pathology Program. Carcasses were necropsied to determine the cause of death, and to identify any contributing pathologies. Mortalities were classified into the categories, predation, collision, disease and unknown.

### Analyses

#### Survival estimates

We estimated annual survival of cats with the staggered entry modification to the Kaplan-Meier survival estimator [13]. Survival distributions were determined by month. Annual periods extended from March to the following February each year, such that survival data were collected from March 2008 to February 2010. Cats that disappeared or dispersed from the study area were right censored during the month they disappeared. We assumed that survival probabilities were independent among individual cats, and that survival probabilities were constant during monthly intervals. Additionally, we calculated survival estimates separately for each sex, and used a Z-test to compare annual survival estimates between sexes.

#### Home range estimates

We used the Animal Movement Extension [14] for ArcView 3.2 Geographical Information System (GIS) software (Environmental Systems Research Institute, Redlands, California, USA) to plot 95% minimum convex polygon (MCP) and 95% fixed kernel (FK) home-range estimates. We also estimated home ranges using an adaptive local convex hull kernel method (LoCoH [15]). We calculated 95% and 50% contours using the adehabitatHR package [16] in the R statistical program (R Development Core Team 2011 v.2.12.2, Vienna, Austria). The maximum distances between two points were used as the *a* values as recommended by Getz et al. [15]. We calculated annual home ranges for each cat that had a minimum of 30 radiolocations recorded during an annual period (the minimum number of locations that spanned more than one season within an annual period). Some cats were monitored in both years and had sufficient location for estimates in both years. We compared mean home range estimates between sexes and reproductive status with ANOVA, and used Pearson’s correlation coefficient to assess the possible relationship with body weight. We estimated home range size with a variety of models that represent historical use (i.e. MCP) as well as more recent models (i.e. LoCoH) for comparison with other studies.

#### Habitat selection and spatial overlap

At some of our sites, coyotes also were monitored as part of a long-term, concurrent study [10]. Coyotes have been monitored continuously since 2000, and capture, handling, and radiotracking methods for coyotes were described in detail elsewhere [17]. As part of this long-term study, radiocollared coyotes were monitored at the Max McGraw Wildlife Foundation, Poplar Creek, and SVNC sites concurrent with cats at those sites. Within each of these sites, we focused specifically on a subset of radiocollared coyotes that had home ranges (100% MCP) that overlapped radiocollared cats. As with cats, we estimated home ranges for those coyotes with >30 locations in a year.

We used the LoCoH home range estimator to assess home range overlap for cats and coyotes that were monitored concurrently on site. This estimator provided the most conservative estimates of home range area [15], and consequently of spatial overlap, among the home range models. Because coyote home ranges were usually considerably larger than cat home ranges, and we were specifically interested if cats avoided areas used by coyotes, we assessed spatial overlap by calculating the proportion of each cat 95% home range overlapped by a coyote 95% home range. To further assess overlap of activity centers, we also calculated the proportion of each cat’s 50% core area that overlapped with a coyote’s 50% core area. We pooled results across study sites, and considered individuals monitored in both years as independent observations if the individual for the other species differed between years. We calculated individual mean overlap for each cat if they were monitored concurrently with >1 coyote, and then an overall mean overlap for 95% and 50% contours.

For those coyotes and cats with overlap, we also assessed whether they differed in habitat use and selection. We were specifically interested in whether the species differed in their use of natural habitats and urban, developed habitat types. We used a land-use type coverage with 28.5 m resolution from 1997 Chicago Wilderness/NASA Landsat Thematic Mapper images for use in ArcView GIS software [18]. We used Gehrt et al.’s [17] reclassification scheme in which the original 164 Landsat categories were reclassified into 8 broad land cover types: However, our interest was specifically focused on patterns of use of natural habitat and urban (residential) land use types. Therefore, we further grouped the land use classes into Urban Land, Urban Grass, Natural habitat, and Woods. Urban Open Space was renamed Urban Grass, and Forest was renamed Woods. High Density Urban, Medium Density Urban and Low Density Urban classes were combined to form Urban Land. Wetland, Surface Water, Agriculture and Barren were combined to form Natural Habitat.

We used a Euclidean distance-based approach [19] to determine habitat selection for each species. For each cat or coyote with greater than 30 locations in an annual period we calculated the distance from each location to each habitat type using ArcMAP 10 (ESRI, Redlands, CA). A number of random locations equal to the number of locations in an annual period were generated for each individual. Random locations were generated inside a 95% MCP based on the pooled locations of conspecific individuals at a study site. We calculated distances from each randomly generated point to each habitat type. Mean distances were calculated for both actual and random points for each individual, and a selection ratio of mean distance from actual locations divided by mean distance from random points was generated for each habitat type and individual. A multivariate analysis of variance (MANOVA) was used to determine whether mean selection ratios differed from 1 to determine the presence of habitat selection. Analysis of variance (ANOVA) tests were used to determine whether individual habitat types were used disproportionately. P-values were adjusted using Holm’s correction to control family-wise error rates.

### Ethics statement

Handling and capture of coyotes was approved by the Ohio State Unversity Animal Care and Use Committee, protocol #2010A00000113. Handling and capture of cats was approved by the Ohio State University Animal Care and Use Committee, protocol #2010A00000114. All trapping and handling protocols followed guidelines of the American Society of Mammalogists (Animal Care and Use Committee) [20]. Permits and permission to work at Poplar Creek Forest Preserve, Beverly Lake Forest Preserve and Ned Brown Forest Preserve were provided by the Cook County Forest Preserve District. Permits and permissions to work at Pleasant Valley Conservation Area and Prairie View Conservation Area were provided by the McHenry County Conservation District. Permission to work at Spring Valley Nature Center was provided by the Schaumburg Park District. Permission to work at Max McGraw Wildlife Foundation was provided by the Max McGraw Wildlife Foundation. No endangered or protected species were involved in this study.

## Results

We captured 43 cats between February 14 2008 and September 11 2009. Our sample was dominated by adult cats with a nearly even sex ratio: 18 adult females, 2 juvenile females, 19 adult males, and 3 juvenile males. The number of cats captured by site ranged from 1 to 11 (Beverly Lake 4, Coral Woods 1, Max McGraw Wildlife Foundation 11, Ned Brown Forest Preserve 1, Pleasant valley 10, Poplar Creek 4, Prairie View 5, SVNC 7) (Figure 1). Twenty-one percent of males and 28% of females had been sterilized prior to capture, 5 females were obviously pregnant or lactating, and 3 females had been reproductively active earlier that year. The majority of sterilized cats came from a single site (6 cats associated with SVNC).

**Figure 2:**
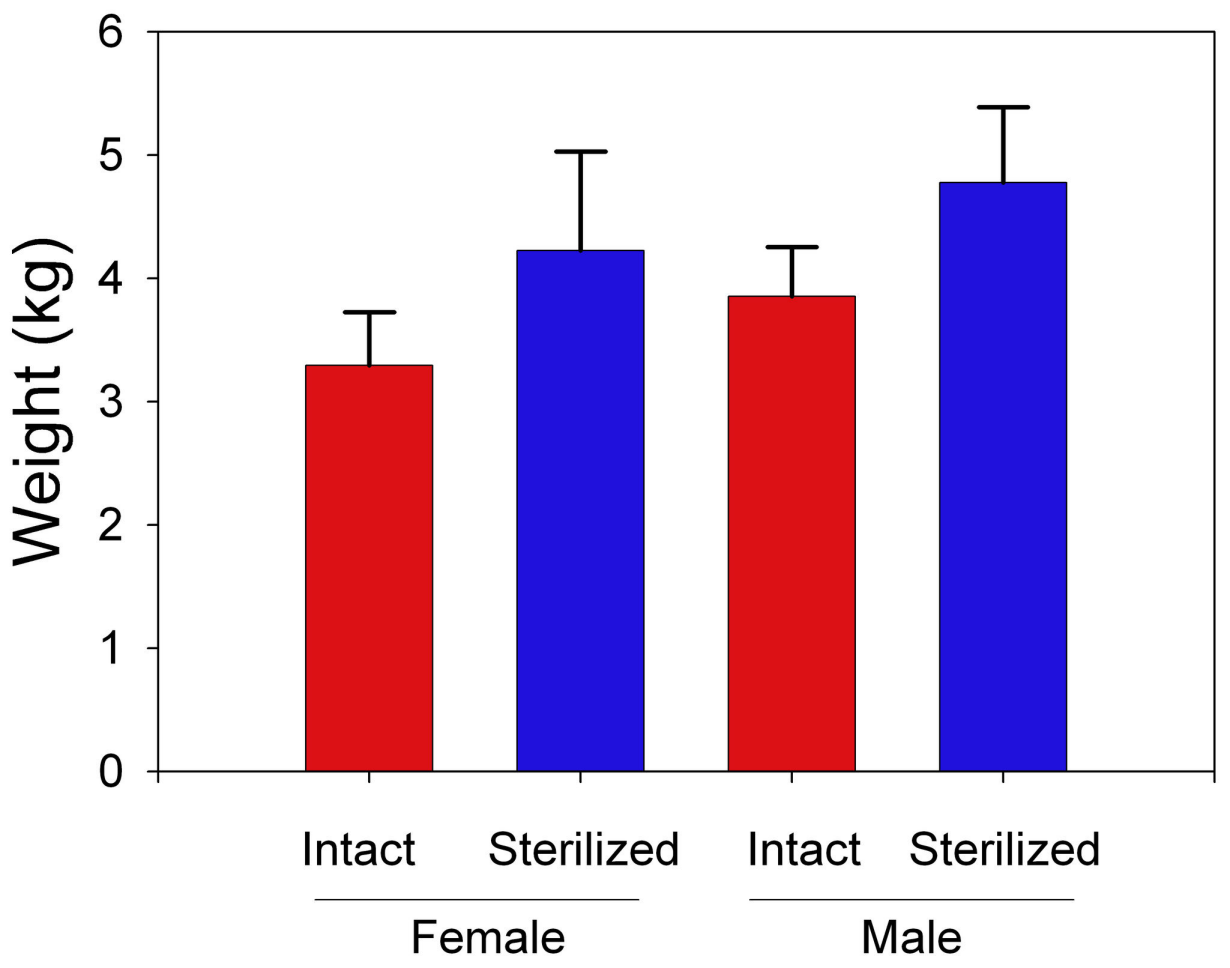
Mean (SE) body weights for free-ranging cats (n = 43) partitioned by reproductive status. All cats were sampled from the Chicago metropolitan area during 2008-2009. Reproductive status was defined as either intact or sterilized.

Mean body weights varied by sex (F_1,30_ = 4.911, P = 0.034) and by reproductive status (F_1,30_ = 14.010, P <0.001). On average, adult males weighed more than adult females for either reproductive group (Figure 2). For both sexes, mean body weights were higher for sterilized cats than for intact cats.

We collected 31 blood samples for serology from initial captures and recaptures of >6 months between captures. We detected low rates of seroprevalence for the two pathogens detrimental to cats, FeLV (0%) and FIV (3%). Likewise, antibody response to feline heartworm (6%) also was detected at relatively low levels. Seroprevalence for *T. gondii* was low for IgM (3%) but over half (58%) of the samples were positive for an IgG response.

### Survival and Causes of Mortality

Of the sample of 43 captured cats, we fitted radiocollars on 39 (of those not radiocollared, 1 was extremely sick at capture and euthanized (pathogen could not be identified), 2 were presumed to be pets, and 1 was too small for a radiocollar). Among radiocollared cats, 20% died during the study, 23% were removed from the system by cat advocates opposed to our research (abduction), 28% were adopted or legally removed, transmitters expired for 13%, and 10% were missing before the end of the study. Cats may have gone missing due to dispersal, a mortality event that also impacted the transmitter (i.e., vehicle collision), abduction, or a resident adopting them without contacting us. However, we were reasonably confident that missing cats were not due to dispersal because we expended considerable effort, including flying, to recover the signals from lost cats. We were able to confirm transmitter expiration by observing collared individuals in the course of radio-telemetry while being unable to hear a signal. As part of the telemetry effort on this project we surveyed roads for dead animals on a regular basis. Among the ‘adopted’ class, three cats were apparently owned cats and radiocollars were promptly removed by owners. Thus, these owned cats were excluded from survival and movement analyses.

The overall annual survival estimate (SE) for free-ranging cats was S = 0.70 (0.10), and estimates did not differ (P > 0.05) by sex (M=0.70 (0.17), F=0.67(0.12)). Of the 8 mortalities associated with radiocollared cats, 1 was because of disease, 2 to vehicle collisions, 3 to predation (apparently coyote), and 2 were because of unknown causes. Disease also was the reason 1 cat had to be euthanized and could not be radiocollared. Predation was attributed to coyotes based on examination of puncture marks on cat carcasses, sign and tracks left around kill-sites and location of kill sites. Feral dogs were not observed in the study area.

### Home Range

Overall, we recorded 2,646 locations, including 1,512 locations in 2008 and 1,134 locations in 2009. We recorded a sufficient number of locations to estimate 36 annual home ranges for 26 cats. For cats with home range estimates in both years, MCP home ranges between years were correlated (n = 10, r = 0.762, P = 0.0104), and FK home ranges were marginally correlated (n = 10, r = 0.569, P = 0.0863). Thus, we reduced the dataset to only 1 estimate for each cat by eliminating the estimate utilizing the fewest locations. Further, from the reduced dataset, MCP estimates were highly correlated (n = 26, r = 0.979, P <0.001) with FK estimates, so we restricted comparisons between groups to FK estimates.

**Figure 3:**
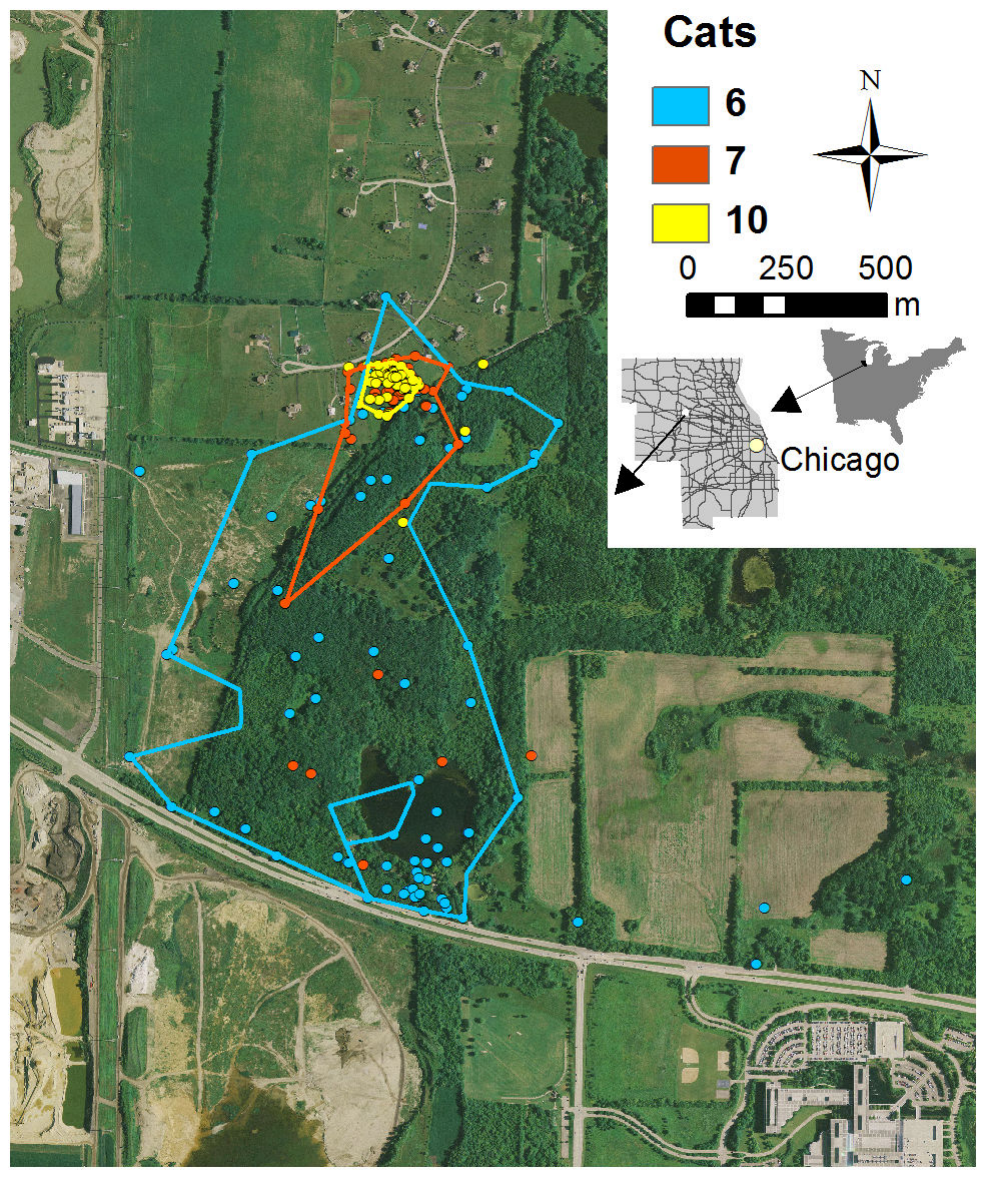
Annual home ranges (95% Local Convex Hull) of free-roaming cats. Relocations occurred during 2008 in the Chicago metropolitan area, and illustrates the individual variation in home range size among cats using the same area. Grey lines on the county map indicate roads.

Mean home-range size for males was larger (F_1,22_ = 6.72, P = 0.017) than mean home-range size for females (Table 1). However, there was considerable individual variation in size of home range within sex classes regardless of the home range model (Table 1). As expected, LoCoH estimates were lower relative to MCP or FK estimates. There was no relationship between home range size and reproductive status (F_1,22_= 0.538, P = 0.471), or with body weight (r = 0.01, P = 0.954). The individual variation in home range size occurred within sites, among cats that presumably had access to the same feeding sites (e.g., Figure 3). Those cats exhibiting extensive movements within habitat fragments were typically adult males, but most males had home ranges similar in size to females’. 

**Table 1:** Summary of mean (SE) estimates of home range size (ha) for free-roaming domestic cats during 2008-2009 in the Chicago metropolitan area.

		MCP			FK			LoCoH	
Sex	N	Mean	Range		Mean	Range		Mean	Range
Male	14	73.8 (19.5)	4.9-198.3		96.0 (27.5)	4.9-290.9		32.0 (9.1)	1.4-93.4
Female	12	30.2 (7.3)	1.3-80.9		29.4 (8.3)	1.4-102.3		8.9 (2.2)	0.6-23.3

Estimators are the 95% minimum convex polygon (MCP), 95% fixed kernel (FK), and 95% local convex hull (LoCoH) home range models.

### Habitat selection and spatial overlap

During 2008, 33 radiocollared coyotes used all or portions of study sites with cats (7 MMWF, 20 PC, 6 SPNC), and 30 in 2009 (5 MMWF, 19 PC, 6 SVNC). Of those samples, 17 coyotes were monitored concurrently with, and used the same portions of the sites with some degree of overlap, with 13 cats from our study (1 cat had sufficient data for 2 years, and was represented twice in the dataset, but with different combinations of coyotes). Mean (SD) home range size for these coyotes was 264 (173) ha for 95% LoCoH kernels, and 54 (41) ha for 50% LoCoH kernels. The larger coyote home ranges extensively overlapped individual cat home ranges at the 95% contour (mean 74%, SD=27%, n=32). However, 50% core areas of cats averaged only 14 (30)% overlap with coyote core areas. Overlap of core areas was typically less than 14% because this mean was skewed by a single cat, cat 41, whose core area was entirely encompassed by a coyote’s core area. Cat 41 was eventually depredated by coyotes.

**Figure 4:**
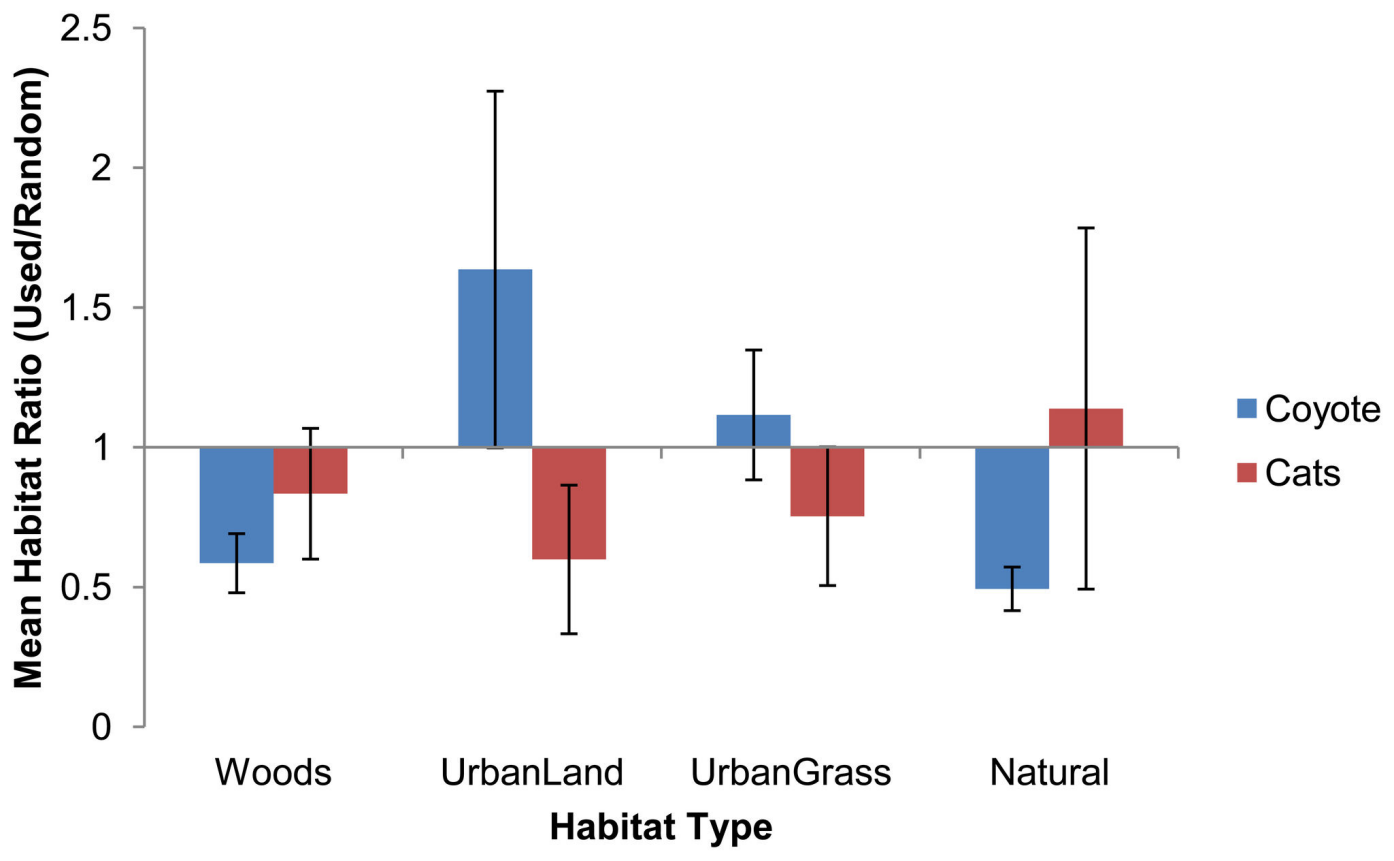
Mean (95% CI) habitat selection ratios for coyotes and free-roaming cats in the Chicago metropolitan area during 2008-2009. Selection ratios are the ratio of the distance to habitat type for observed locations/random locations. Ratios <1 indicate selection for the habitat, and >1 avoidance.

**Figure 5:**
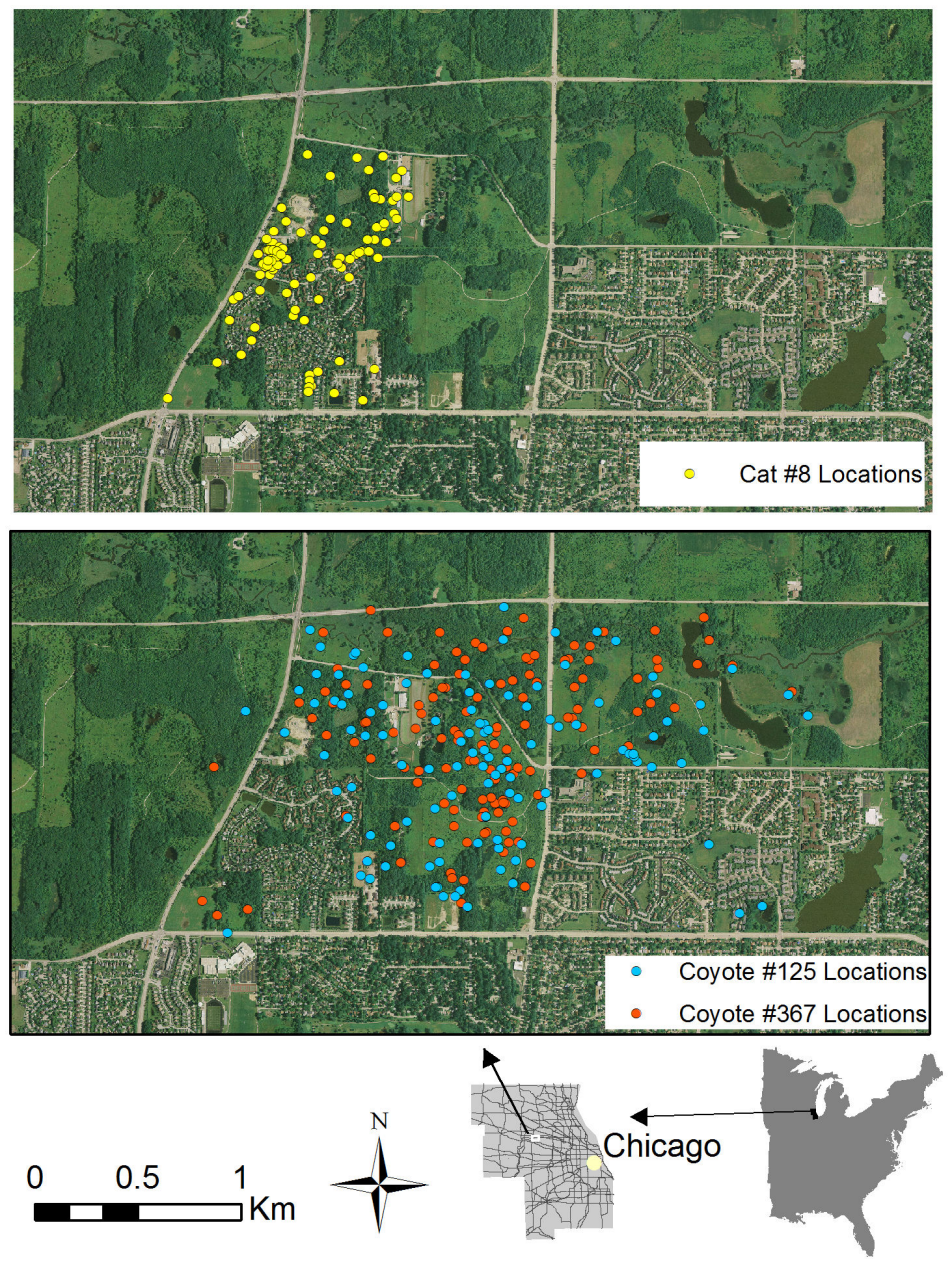
Spatial overlaps between cats and coyotes using the same urban park. Radiolocations for a free-roaming cat (left panel) and two coyotes (right panel) exhibiting spatial overlap, during 2008 in the Poplar Creek Forest Preserve, within the Chicago metropolitan area. Cat locations were closely associated with urban development, and did not occur in areas with high coyote activity. Grey lines on the county map indicate roads.

Coyotes and cats differed in patterns of habitat selection. Cats exhibited significant selection for Urban Land (adjusted P = 0.0261), and no selection for other habitat types (adjusted P’s > 0.18). In contrast, coyotes exhibited selection for Woods (adjusted P <0.001) and Natural (adjusted P <0.001), with no selection (adjusted P’s > 0.12) for urban habitat types. Patterns of selection ratios, even if nonsignificant, differed between species (Figure 4). For example, the mean cat selection ratio for Natural habitat was >1 (indicating avoidance), although the variation was substantial, due to a minority of cats that extensively used natural habitats. Coyote mean selection ratios for urban habitat types were consistently >1, especially for urban land (Figure 4). In general, cat locations were closely associated with human areas and not with areas with high coyote activity (e.g., Figure 5)

## Discussion

In general, we found that free-ranging cats were in better condition than we expected, with a relatively high survival rate and low exposure to cat-related pathogens, with the exception of *T. gondii*. Although our trapping was conducted in natural habitat fragments, and therefore our sampling was biased toward cats using fragments, our study animals appeared to live primarily at the periphery of natural habitat fragments, which was likely an avoidance of coyotes, as coyotes selectively used natural habitats and avoided developed areas.

There was little evidence that transmissible diseases were important as serology indicated low exposure to FIV and FeLV, which was consistent with the low frequency of disease-related mortality during our study. Although disease appeared to be a minor component of cat dynamics within our sample, there was the potential for cats to negatively impact other species, including humans, through their transmission of *T. gondii* [21]. Exposure to *T. gondii* is common among mesocarnivore species in the Chicago metropolitan area. For example, striped skunks ( *Mephitis* *mephitis* ) were 60% seropositive for *T. gondii* [22], raccoons ( *Procyon* *lotor* ) 38% [23], and coyotes range from 38 to 63% annually (Gehrt unpublished data). Our results for *T. gondii* contrast with Bevins et al. [24], in which those authors reported substantially lower IgG seroprevalence of *T. gondii* for domestic cats in Colorado and southern California, such that native cat species were implicated in the prevalence of *T. gondii*. In our system, native cat species (such as bobcat, *Felis* *rufus* ) are rare or absent; therefore, the prevalence of *T. gondii* is likely the result of domestic cats as host. While the reasons for these regional differences in the role of domestic cat as host of *T. gondii*, are unclear, our results highlight the potential for variation in host/parasite dynamics across different urban systems.

The annual survival rate of free-roaming cats in our study is at the higher end of the mesocarnivore survival spectrum in the Chicago area (Table 2). Most of these species have been studied in the same general location, or area, within the metropolitan area, and in some cases within the same urban parks. Undoubtedly survival of free-ranging cats is lower than owned cats restricted to indoors, but our data indicate that they have an annual survival that is relatively high compared to most other mesocarnivore species, with only the raccoon exhibiting a survival estimate higher than that of the cats (Table 2). The relatively high survival is consistent with the view that the population is largely healthy with few disease issues. Schmidt et al. [25] and Horn et al. [12] reported similar survival estimates for cats with different ownership status, with survival of feral or unowned cats near 50% and survival for semi-feral or owned cats >90%. However, Schmidt et al. [25] apparently did not have coyotes present in their system, and predators were regularly removed from Horn et al.’s [12] study area. Our study area had more extensive urbanization, including road densities and traffic volume, than either of those studies, as well as higher coyote densities.

**Table 2:** Summary of annual survival estimates (S) for mesocarnivores in the Chicago metropolitan area.

Species	Scientific Name	N	S	Years	Source
Domestic cat	*Felis catus*	39	0.70	2008-2009	This study
Striped skunk	*Mephitis* *mephitis*	73	0.41-0.51^a^	1998-2001	[39]
Red/Gray fox	*Vulpes* *vulpes* *, * *Urocyon* *cinereoargenteus*	9	0.49-0.62^b^	2006-2008	[40]
Raccoon	*Procyon* *lotor*	102	0.57-0.88^a^	1995-1998	[41]
Coyote	*Canis* *latrans*	181	0.58-0.70^c^	2000-2006	[10]

All estimates were determined from radiotelemetry data using the Kaplan-Meier staggered entry design. Ranges are presented for those studies with multi-year estimates and/or multiple study sites within the larger urban landscape.

^a^annual estimates across sites and years

^b^upper estimate is the ‘best-case’ model, lower estimate is the conservative, and more likely, estimate

^c^annual estimates across demographic groups

Home range size in our study was similar to previous studies of free-ranging cats from more rural landscapes [12,26], but was considerably larger than mean home range sizes for cats in Schmidt et al.’s [25] study. Some studies have reported sex differences in home range size, and considerable variation in movement patterns among individuals [12,27]. Some of the variability in home range size may be due to ownership and the level of supplemental feeding [12,25]. We were aware of food provisioning to some degree at each of our study sites, but cats varied in the extent of their movements even though they presumably had access to the same food resources (Figure 3).

Free-ranging cats located within the urban natural areas were primarily adults with an even sex ratio, and in generally good physical condition. Most cats were reproductively active. Our sample sizes did not allow us to rigorously test whether sterilization affected annual survival, but we did detect higher body weights for sterilized cats.

### Response to coyotes

Previous studies have provided data suggesting coyotes can influence the distribution of cats in urban landscapes [5]. However, our study is the first to monitor individuals of both species concurrently. Coyotes in our system appear to inhibit cat use of the natural habitat fragments through a combination of predation and cat avoidance of coyote activity. Coyote predation on cats has been reported to a limited extent elsewhere [5,28], although the frequency with which this occurs across metropolitan areas is largely unmeasured. Coyote densities within natural habitat fragments within our study area were relatively high [10]. Minimum densities using only the number of radiocollared coyotes in a site ranged from 1.4-4.0/km^2^ in 2008 [10] and 1.4-14/km^2^ in 2009 (Gehrt unpublished data). By comparison, coyote densities in rural landscapes are typically <0.5/km^2^ [29]. Additionally, coyotes selectively use natural habitat patches and, at our specific study sites, resident coyotes typically restrict their movements within the fragments and avoid residential areas [10,17]. Thus, there was presumably considerable risk to cats within habitat fragments and much less so within the urban matrix, which may have contributed to the relatively high survival rate and low predation rates for cats. Given that coyotes are distributed across the Chicagoland landscape [10], this ‘coyote effect’ limiting cat presence in natural habitat fragments should be prevalent across most natural fragments. Indeed, livetrapping of mesocarnivores across the Chicago metropolitan area reported higher capture rates for cats in the urban matrix than in natural habitat fragments [23].

Our results are consistent with previous studies conducted in areas with potential predators on cats, although specific, concurrent, data on the predators were not available [30,31]. In each of these cases, the limited movements into habitat fragments were attributed, at least in part, to predator avoidance by cats. It is important to note that we observed interspecific differences in habitat use/selection despite restricting our analysis to coyotes that overlapped cats to some degree. Our analyses are conservative and the interspecific pattern of habitat use would likely be more disparate if we had included coyotes located exclusively within the habitat fragments, which was common [17]. Thus, it is clear the two species are largely segregated across the metropolitan area. 

Our results support the notion that the ecological impact of cats in natural habitat fragments is minimized due to interference competition from coyotes [5,32]. Intraguild competition often structures mammalian carnivore communities, either through direct interference, sometimes including intraguild predation, or indirect interference [32-34]. Although our sample is small, it suggests that coyotes may take cats as competitors or as prey, and that they may not be mutually exclusive. Indeed, in a recently-completed study of colony cats within our study area, we recorded at least 11 fatalities of cats from coyotes, of which 9 were partially or wholly consumed, and 2 were not (although one of these was cached; Gehrt, unpublished data). We had no study sites without resident coyotes, therefore we cannot state conclusively that patterns of cat habitat use were a response to the presence of coyotes. However, in studies of cat use of habitat fragments in systems where coyotes were rare or absent, cats were common in the habitat fragments, and Sims et al. [2] reported an order of magnitude increase in density of cats in urban green spaces without large predators. Likewise, cats in rural Australia were observed to select habitat that afforded them protection from sympatric dingoes [35], and on a larger scale, there appears to be a strong negative association between dingoes and free-ranging cats [36,37]. Dingoes represent top predators in Australia, similar to coyotes in our system.

Our results illustrated that, although free-ranging cats are common in the Chicago landscape, their use of natural habitat fragments was limited. Consequently, the ecological impact of cats in those areas, via predation of native species, also was likely limited. However, we do not know the impact of cats within the larger urban matrix, where coyote activity is relatively limited. Interference competition by coyotes on cats may reduce predation rates on native fauna to more closely approximate those of indoor/outdoor cats, who tend to limit their use to the urban matrix [31]. Finally, estimates of the ecological impact of cats, extrapolated over large geographic areas [38], are likely to overestimate the impact of cats if the effects of interference competition by coyotes are not considered.

## Conclusions

Increasing evidence suggests that free-ranging cats limit their use of natural fragments, likely in response to risk of intraguild predation in addition to human activities [30]. Thus, the ecological impact of cats in such areas may be mitigated despite their abundance and proximity, and conservation programs should consider the potential role of coyotes in buffering natural areas from cat activity. However, more research is needed within the urban matrix to determine predator-prey relationships and the impact of cats in developed areas. Although we documented restricted movements by free-ranging cats and little evidence of disease among cats, a potentially negative aspect of free-ranging cats is transmission of zoonoses such as *T. gondii* to wildlife, pets, and humans [21].
